# Pathophysiology of SARS-CoV-2 associated ischemic stroke

**DOI:** 10.25122/jml-2021-0117

**Published:** 2022-01

**Authors:** Josef Finsterer, Fulvio Alexandre Scorza, Carla Alessandra Scorza, Ana Claudia Fiorini

**Affiliations:** 1. Neurology & Neurophysiology Center, Vienna, Austria; 2. Neurological Department, Federal University of São Paulo (UNIFESP), São Paulo, Brazil; 3. Program of Postgradual Studies of Phonoaudiology, Pontifícial Catholic University of São Paulo (PUC-SP), São Paulo, Brazil; 4. Phonoaudiologic Department, Paulista School of Medicine/Federal University of São Paulo (EPM/UNIFESP), São Paulo, Brazil

To the Editor,

We read with great interest the article by Vacaras *et al.* about a 50-year-old male with moderate COVID-19 who experienced an ischemic stroke nine days after onset of COVID-19 [1]. Initially, acetylsalicylic acid and clopidogrel were given but stopped after a second CT scan revealed left frontal subarachnoid bleeding (SAB) and hemorrhagic transformation of the ischemic lesions in the area of the left cerebral median artery [1]. The stroke was causally attributed to the viral infection [1]. The study is appealing but has several limitations, which raise the following comments and concerns.

The first limitation of the study is that no digital subtraction angiography (DSA) was carried out to eventually detect the source of SAB, in particular aneurysm formation, cavernoma, developmental venous anomaly (DVA), acquired arterio-venous fistula, or congenital arterio-venous malformation (AVM). In case of unexplained cerebral bleeding, it is compelling to search for the source of bleeding, particularly in the absence of atherosclerosis or arterial hypertension.

A second limitation is that no explanation was provided regarding the normal cerebrospinal fluid (CSF) investigations despite the SAB. Since the patient experienced a SAB, one would expect xanthochromia and positivity for oxy-hemoglobin, met-hemoglobin, or bilirubin.

The third limitation is that the CSF was not tested for SARS-CoV-2. If ischemic stroke and SAB are attributed to SARS-CoV-2, it is crucial to document the presence of the virus in the CSF. lt would also be interesting to know if the CSF levels of IL-6, IL-8, IL-1beta, and TNF-alpha were elevated as has been previously reported in patients with SARS-CoV-2 associated encephalitis [2].

A fourth limitation is that cardio-embolism was not excluded. Though the patient underwent several ECG recordings, which were not indicative of atrial fibrillation, the morphology of the ischemic lesions on cerebral MRI suggests embolism rather than athero-thrombosis. Thus, the patient should have undergone echocardiography and long-term ECG recordings. Since thrombo-embolism was suspected as the cause of ischemic stroke, intracardiac thrombus formation and the presence of a patent foramen ovale (PFO) need to be excluded. Intraventricular thrombus formation in COVID-19 patients may originate from atrial fibrillation, myocarditis, heart failure, Takotsubo syndrome, all cardiac complications of COVID-19 [3].

A fifth limitation is that the patient was treated with anti-COVID drugs, which are largely ineffective. No beneficial effect of ritonavir/lopinavir or hydroxy-chloroquine has been reported so far. We should know why these drugs were still used. Since these drugs can exhibit severe side effects, the application of these compounds should be avoided [4].

A sixth limitation is that coagulopathy was not excluded. Since COVID-19 can go along with hyper- or hypo-coagulability, it is essential to test the intrinsic and extrinsic coagulation system extensively. Since the initial cerebral CT scan was normal on hospital day-1 and since the patient was treated with a combination of acetylsalicylic acid and clopidogrel, it is also conceivable that bleeding resulted from the double antithrombotic regimen.

The patient was discharged after 7 days, but the cerebral MRI was performed 8 days after stroke onset. Therefore, we should know why the MRI was carried out after and not during hospitalization.

Overall, the report has several limitations which challenge the results and their interpretation. These limitations should be addressed to substantiate the conclusions.

## Acknowledgments

### Conflict of interest

The authors declare no conflict of interest.

### Authorship

JF was responsible for the design, literature search, discussion, first draft, critical comments, and final approval. FS, CS, and AF were responsible for literature search, first draft, discussion, critical comments, and final approval.
